# Health Promotion and Obesity in the Arab Gulf States: Challenges and Good Practices

**DOI:** 10.1155/2019/4756260

**Published:** 2019-06-09

**Authors:** Anastasia Samara, Pernille Tanggaard Andersen, Arja R. Aro

**Affiliations:** Unit for Health Promotion Research, University of Southern Denmark, Niels Bohrs Vej 9-10, Esbjerg 6700, Denmark

## Abstract

This debate paper focuses on available strategies, policies, and challenges of health promotion for combating obesity in the Arab Gulf states (Saudi Arabia, Bahrain, Kuwait, Oman, and Qatar). The paper focuses on the abovementioned countries due to their similarity on many aspects and because of their alarming obesity rates that are on the rise and keep increasing. The paper argues that there are significant efforts to be made in sectors such as policies, intersectoral work, primary healthcare, health promotion strategies development, and qualified personnel for health promotion and health education. Among the six states, Qatar, United Arab Emirates, and to a degree Oman have shown some development with regard to the implementation and evaluation of obesity-related health promotion policies, and thus other Arab Gulf countries could be inspired by existing good practices and move from good intentions to using their available wealth to invest in the implementation and evaluation of published policies and strategies. All Arab Gulf countries are in need of more qualified personnel and the development of infrastructure that can help tackle the growing obesity challenge that such countries are experiencing.

## 1. Obesity Prevention in the Arab Gulf States

The six Arab Gulf states are among the countries that have very high levels of obesity and overweight individuals. Around 30% or more of the population in these countries is obese (>or = to 30 kg/m^2^), and more than 60% have a weight range higher than normal (>or = to 25 kg/m^2^). Similar obesity and/or overweight levels are found also in other Middle Eastern countries, in the Maghreb countries, and also in the United States, New Zealand, Canada, Australia, and in some European countries [1].  Table 1 shows the prevalence of overweight and obesity for males and females in the Gulf States for 2016 [1]. It also shows the population for each country based on the latest estimate or census [2–7].  Table 1 also shows the prevalence of type 2 diabetes by gender. The highest rates are seen in KSA, Kuwait, and Qatar, and they are similar (more than 10%) to other Eastern Mediterranean countries such as Lebanon, Turkey, and Egypt as well as Morocco, Algeria, and Tunisia but are higher than those rates recorded in the USA, Australia, or UK [8].

The alarming levels of obesity and overweight have awakened the Gulf countries to develop country- and community-level health promotion strategies in the last few years. For example, these kinds of strategies have been in place in the Nordic countries [9, 10] in Australia and New Zealand [11] and in Switzerland [12] for more than ten years.

The aim of this debate paper is to present and discuss the main challenges that the Arab Gulf states face in relation to health promotion and obesity. This debate is particularly important because of the rise of the obesity epidemic in these countries. Important aspects in health promotion are policy development, building up intersectoral work, establishing primary healthcare centers as settings [13], developing workforce capacity in health promotion as well as prioritizing health education, and health promotion at leadership and governance [14].

## 2. What Are the Main Challenges for the Arab Gulf States?

The Arab Gulf states share similar cultural, political, and geographical characteristics. They are all wealthy states with high standards of living (GDP for 2016 ranging from 32.179 billion for Bahrain to 646.438 billion for Saudi Arabia) [15] that have been particularly exposed to and influenced by the western way of life, including eating habits (fast food and processed food) and physical inactivity (ways of commuting, working environments, and types of work). A study showed that physical inactivity, for example, in Saudi Arabia is not related to sociocultural factors but rather to lack of facilities for women to practice sports [16]. On the contrary, the Arab Gulf states are also quite different in terms of size, population, and governance from a centralized system in Saudi Arabia, to a decentralized system in Oman, and to local governance, like a city state in all other Gulf states due to their small size and population.

It is very common that health promotion is seen/interpreted as awareness campaigns and that dietary choices as well as physical activity are purely individual choices. The dominant concept worldwide has been that of risk, and the individual is put in the position of personal responsibility to reduce this risk [13]; the Arab Gulf states are no exception to this interpretation. However, there are other wider social and environmental determinants for obesity such as marketing, taxes, pricing, and availability of healthy food, physical activity facilities, and cultural traditions. Only targeting individuals and neglecting these wider, social, and ecological determinants of behaviors and consumption is not sufficient to curb the obesity epidemic in the Arab Gulf states. This debate paper identifies central areas of challenges, including policies, intersectoral work, the role of primary healthcare, and lack of qualified staff, which all require further development in the Arab Gulf countries to curb the obesity epidemic. A more elaborate work on the strategies and policies of the Gulf states will be presented in a future book chapter [17].

### 2.1. Policies

Policies are an integral part of health promotion, and some policies relating to school environments [18], food labeling [19], and others are very important for combating obesity. Evidence also exists that catering services in schools and workplaces contribute to healthy eating habits in the population [20]. Good examples of healthy meal policies implemented in schools and workplace canteens come from Sweden and Finland [9, 20, 21].

All Arab Gulf states have made some efforts with regard to prevention and health promotion policies and meant implementation such as (1) soft drink taxation adopted by Saudi Arabia [22] and United Arab Emirates [23], (2) banning soft drinks and junk food in hospitals in Qatar [24], (3) workplace health promotion in Qatar [25] and United Arab Emirates where it mostly focused on government entities [26], and (4) nutrition and/or physical activity at the school setting in Qatar [27], United Arab Emirates [28–30], Bahrain [31], and Oman [32]. Qatar and United Arab Emirates are the most advanced when it comes to developing and implementing policies at the school setting, e.g., when it comes to introducing physical education courses for all schools [33, 34].

However, most of the Arab Gulf countries lack important local policies in schools, for example, the availability of healthy food and beverages in school canteens, regular screening for obesity and overweight, and extracurricular activities. In addition to the above, work environment policies and food labeling policies are needed. Saudi Arabia showed an example by introducing a mandatory regulation to mark calories in all restaurant menus as of 2019 [35]. Food labeling both in restaurants and (packaged) food items for sale is urgently needed for the population to make informed food choices. Furthermore, the Arab Gulf countries could also establish a food database for the use of all citizens in line with the Finnish food database [36].

### 2.2. Intersectoral Work

Intersectoral work is the backbone of health promotion and for applying the principle of “Health in All Policies.” This strategy aims to include health considerations in policymaking across different sectors that influence health, such as transportation, agriculture, land use, housing, public safety, and education [37]. “Health in All Policies” is adopted in Nordic countries [9], in Australia [38], and in the Netherlands [39].

The approach “Health in All Policies” has been initiated in Oman [40], United Arab Emirates [41], and Qatar [42] which has involved the collaboration of the Ministry of Education, Municipalities, academia, sports councils, etc. A clear example is the development of the National Healthcare Strategy 2011–2016 for Qatar as a common effort of many stakeholders (Ministry of Interior, Supreme Council for Family Affairs, and Permanent Population Committee) [42]. However, the Oman strategy is still in the implementation stage.

On the contrary, Saudi Arabia, Bahrain, and Kuwait do not show established intersectoral work. These countries often do not mention intersectoral work in their national healthcare strategies [43–45].

In light of the current situation of the Arab Gulf states, it is time for them to introduce the “Health in All Policies” approach to create dynamic interactions across different societal sectors and on different levels of the society (national, regional, and local) to create the infrastructures and environments which could enable healthy choices and an active lifestyle for citizens.

### 2.3. Primary Healthcare

Primary healthcare should be the main healthcare entry point for citizens and also a major actor in promoting health and evaluating the status of the population. Primary healthcare contributions have shown positive results in obesity reduction [46, 47], for example, Finland with its Health Care Act of 2010 that included health-promoting activities as part of primary healthcare purposes [48] and Canada with many examples of health promotion incorporated in primary healthcare [49].

Primary healthcare is not yet established as an entry point for all Arab Gulf states. In addition, in most of them, the primary healthcare centers do not even play a significant role in health promotion, and future plans for them in current healthcare reforms (all Arab Gulf states are going through them) do not suggest enhancing this role [43, 44]. Qatar is the only example that puts primary healthcare at the forefront of action for health promotion [25]. In Abu Dhabi, primary healthcare centers have facilitated the screening of the population for noncommunicable disease risk factors (Weqaya program) [50]; however, they do not have any further roles and tasks in health promotion.

Definitely, some of the Arab Gulf states such as Oman and Saudi Arabia have different priorities for primary healthcare since they strive to ensure basic services before even considering health-promoting activities [40, 43]. However, with the increasing obesity problem, the above argument is no longer sufficient for neglecting these services. There is a need for primary healthcare in both countries to increase awareness of structural health promotion, including disease prevention strategies, the policy level, and contextually salient and setting-based interventions.

### 2.4. Qualified Personnel and the Specialization Issue

Appropriate human resources are crucial in fulfilling tasks and functions related to health promotion in the primary care sector and in the health sector in general [13]. Examples of countries with highly qualified health promotion professionals are the USA [51] and Canada [52].

A common problem for all of the Arab Gulf states is the current limited number of specialized personnel in health promotion and health education especially for Saudi Arabia that needs to cover a big population (32,612,641 for 2017) [2]. In general, healthcare is lacking competent and qualified personnel that are nationals and sufficient in numbers to cover current needs. Such states majorly depend on foreign specialists for different areas. For example, Saudi Arabia requires foreign staff for all specializations in public health due to its size [53], whereas Kuwait is lacking professionals mostly for policy, strategy, and plan development as well as in the fields of nursing and dentistry [414]. Bahrain [54] and Oman [55] are exceptions in their capacity for public health professionals (such as nutritionists and health educators although not health promoters).

With regard to health education, there are some bachelor-level health education programs, e.g., in Saudi Arabia [56, 57], but there is a clear need for more focused and prioritized university departments/units/curricula for health promotion/health education at the national levels in the Arab Gulf States. One solution is to offer tailor-made professional level courses in evidence-based, multilevel, contextual health promotion interventions.

Training professionals will require time and resources but would be very important in building the needed competences and infrastructure for health promotion to comprehensively tackle the regional obesity challenge.

### 2.5. National Programs and Action Plans

National obesity programs with concrete action plans and follow-ups are needed.

A solid national strategy for nutrition/physical activity/health promotion with specific goals, targets, actors involved, procedures, implementation, and evaluation processes is needed to create changes in health promotion and obesity [14]. Examples of national programs related to obesity exist from different countries such as France [58] and UK [59].

All Arab Gulf states have developed at least one document on health promotion that focuses on nutrition and physical activity. However, with the exception of Qatar [41] and Abu Dhabi [50] that have several concrete strategy documents, all other countries only have issued documents that are limited with time-specific goals, ways of follow-up, tangible expected outcomes, specific procedures, etc. This makes the efforts of each country somehow arbitrary and not enough focused on specific actions/programs.

Implementation and evaluation are very important for health promotion strategies as they are for any strategy. The major problem in most cases in the Arab Gulf states is that the strategies are often not implemented or followed up, and also health educators and heath promoters often are not at the backbone of these strategies. Due to these two factors, the strategies remain good intentions with little value even if they are well articulated and developed on paper. There is a need to establish an institutional system, which links intersectional work, prioritizes tasks, and gives a framework for health promotion initiatives at different levels and the possibility to develop standards for implementation and evaluation. For this kind of system to function cost-effectively, accountability must be assigned to certain actors.

The Arab Gulf states need to consider seriously the development of such programs and action plans with the support of policymakers, health promoters, and other professionals that are experts in different fields related to health. One way of addressing this challenge can be through providing more significant budgets for health promotion programs and employing experienced (foreign or local if available) professionals. In the same direction, it is important to strengthen the presence of health promotion departments that are often nonexistent and the presence of health promotion in departments of other ministries as a part of a “Health in All Policies” approach.

## 3. Conclusion

For the Arab Gulf states, the biggest challenges are as follows: (1) the development of substantial health promotion strategies, especially their implementation and evaluation and the administrative structure around health promotion, (2) the availability of national competent professionals for health promotion, and (3) the development of multilevel and intersectoral work and in general applying a “Health in All Policies” approach. These are important challenges that require serious consideration because they are at the root of developing a solid and consistent health promotion strategy to combat the challenges of obesity. Qatar and the United Arab Emirates provide examples of some efforts in that direction but need more focused and elaborate work and a more institutional system to create sustainability. On a final note, the Arab Gulf countries need to focus their efforts more towards these aspects of their health strategy or else will face the increasing costs of diabetes care and the increase of other chronic conditions related to obesity more and more in the coming years.

## Figures and Tables

**Table 1:** General characteristics of the Gulf states.

	KSA	Kuwait	Bahrain	Qatar	United Arab Emirates	Oman
Population	32,612,641 (2017)	4,132,415 (2016)	1,423,726 (2016)	2,700,539 (2017)	9,121,167 (2016)	4,634,812 (2017)
Surface area (square km)	2,149,690	17,188	774	11,628	77,700	309,500
Obesity prevalence (male) 2016	31%	33%	26%	33%	28%	23%
Obesity prevalence (female) 2016	42%	46%	37%	43%	41%	34%
Overweight and obesity prevalence (male) 2016	68%	72%	64%	71%	66%	61%
Overweight and obesity prevalence (female) 2016	72%	75%	69%	73%	71%	66%
Type 2 diabetes prevalence (male) 2016	15%	15%	9%	13%	8%	7%
Type 2 diabetes prevalence (female) 2016	14%	15%	8%	13%	9%	8%
